# The Pennsylvania Journal of Prison Discipline and Philanthropy

**Published:** 1845-09

**Authors:** 


*The Pennsylvania Journal of Prison Discipline and Philanthropy.* Vol. 1, No. 3. July, 1845.

The books, whose titles are at the head of this article, appear to be of dissimilar character; yet they all tend more or less to the improvement of public or private hygiène, or of both. The treatise of M. Levy is intended to embrace the whole subject of hygiène. The Reports of the Commissioners for inquiring into the state of large towns and populous districts is a most valuable contribution to public hygiène. The work of Dr. Metcalfe exhibits a wide field of inquiry, which he has tilled with zeal and ability, in a manner, indeed, which is creditable to him, in several respects, as a man of medical and general science, and in many of its parts, matters of deep moment to the health of the individual and of society are canvassed; whilst the Pennsylvania Journal of Prison Discipline and Philanthropy, as its title imports, has a more restricted range, unless, indeed, we take philanthropy in its expanded sense, and then it includes every thing that may tend to the well-being and happiness of man. Hygiène, however, is an important object with the "Journal;" and it inquires freely and industriously into everything that can ameliorate the hygienic condition of the outcast from society, or of him who is suffering under the direst of all calamities—mental alienation.

We have "many a time, and oft," urged the importance of a greater attention to hygiène. Our Boards of Health in the cities have directed their best energies to urgent matters of public hygiène; but their ordinances have been mostly confined to the removal of nuisances which offend the senses, and which would give occasion to popular outcry were they suffered to persist,—or to the enforcement of quarantine or similar sanatory laws of very questionable expediency, and calculated to fetter commerce—often most unnecessarily. The great and momentous subjects of the physical causes of high rates of mortality in particular localities—the general sanatory condition of cities—the different rates of mortality and births amongst various classes in the same district, and in urban as compared with suburban districts; amongst manufacturers, compared with agriculturists; in drained and in undrained districts, &c.; the supply and filtration of water; the structure of the streets and buildings; the cleansing of streets and houses, and the application of refuse; the causes of disease in cities, and the remedial measures to be adopted,—all of which have occupied the protracted attention of the English Commissioners, have rarely received a passing attention from our Boards of Health, useful as such boards have unquestionably been.

The two Reports of the Commissioners, now before us, are monuments of energy and ability admirably directed. All modern contributions to public hygiène—even those of the Parisian authorities—are indeed cast into the shade by those, which, of late years, have emanated from the Registrar-general of England, from Mr. Chadwick, and from the various commissioners appointed to examine into the sanatory condition of the *industrial* and other classes; and of these, not the least important, in size, and in the value of their contents, are the Reports now under notice. Most elaborate investigations were made by the commissioners themselves; competent professional and other persons were examined where light was to be expected from their testimony; and no stone has been left unturned to discover the sources of disease, and the best means of preventing it. In testimony of the wide sphere of the labors of the commission, we may state that the appendix, amongst other matters, contains a full account of the "watering" of the cities of Philadelphia and

New York, and the answers to the questions of the commissioners in regard to the former city having been furnished by Mr. Strickland, the architect and engineer, to whom the commissioners make due acknowledgments.

It would be utterly impossible for us to analyze a work of this nature. In place of such a futile attempt we shall satisfy ourselves with the following extract from the "Second Report."

After stating, that Registers of Deaths are generally the best guide in such inquiries, the commissioners observe:

"As the subjects specified in your Majesty's commission are essentially of a practical character, we have endeavored to avoid as far as possible the discussion of the theoretical causes of disease. All the medical witnesses examined before us are unanimous as to the injurious effects produced by emanations from animal or vegetable matter in a state of decay, whether they act as direct or contingent causes of disease; and they are quite concurrent in their opinion, that the existence of such causes and their prevalence have been sufficiently ascertained to require the interference of the legislature. The presence of such emanations, whether they be derived from stagnant ditches, from open cesspools, or from accumulations of decaying refuse, is a great cause of disease and death, not confined to the immediate district in which they occur, but extending their influence to neighbouring, and even to distant places. These physical causes of disease may affect various localities and different classes of persons, but are most common and virulent in the neglected districts and dwellings of the poor, who are peculiarly exposed to the aggravating influences of such causes,—not necessarily connected with their condition in life, but capable of being removed by efficient drainage, cleansing, improvements of buildings, ventilation, and a sufficient supply of good water. It is too commonly supposed, that the evils above adverted to are the inseparable concomitants of poverty; and, doubtless, so long as the inhabitants of the most neglected and filthy abodes in crowded cities are unable to provide for themselves better and healthier dwellings, sufficient light and air, more open situations, effective cleansing and drainage, and adequate supplies of water, their vigour and health are undermined, and their lives shortened by the deleterious external influences consequent upon the want of efficient arrangements for securing the above objects. The operation of general sanatory arrangements will enable a greater number to contribute a share to such arrangements, by which they must largely benefit, and thereby, and at a comparatively small cost to the community at large, have the advantage of the remedial improvements above specified.

"Without entering into any discussion upon the influence which poverty and distress may occasion on the rates of mortality, which no sanatory improvements can entirely prevent, we are desirous to remove the injurious impression that a great amount of excessive disease and death in this country is due to causes which cannot in a considerable degree be removed by legislative enactment, when earnestly enforced. At the same time, we must express our opinion that the efficient execution of the law will tend to reduce sickness and disease, and so far increase the means of the poor. Medical witnesses of much experience state that the continued action of injurious emanations, though they may not always produce fever, often become the cause of some of the most common and fatal maladies of this country, and the residence more or less prolonged in a vitiated atmosphere is a great cause of the scrofulous diseases, extensively prevalent in the large towns. In an enquiry into the influence of employments on health, it appears that the relative excess of deaths from consumption among tradesmen and artisans, compared with other classes, is mainly to be attributed to the vitiated state of the atmosphere in their shops and dwellings. The average age at death from consumption has been found to be lower in the case of tradesmen than among artisans; this is stated to be owing to a larger proportion of the latter being employed in out-door work, and therefore less continually exposed to the influence of an impure air. In addition to the evils arising from the absence of ventilation in the interior of dwellings, a great amount of disease is engendered by the polluted condition of the atmosphere in the close and confined courts in which a large proportion of the poor constantly dwell.

"Our attention has been called to the consideration of the ages at which the physical causes of disease produce their most marked effect; and while the returns show, that these effects are peculiarly severe on infantile life, yet they are not confined to any particular age, acting powerfully on persons in the full vigour of life, as well as on the younger part of the population. These returns all show, that the extreme pressure of the physical causes of disease is upon the working population, shortening the average duration of life from 1 to 20, and even to 30 years, and decreasing to a material extent the working ability of the survivors. We find, however, at the same time, that the duration of life of the middle and higher classes is materially lessened by the pressure of these removable causes of disease. In the diseases which follow, in a more marked degree, from the direct or indirect influence of injurious emanations, especially in the case of fevers of the typhoid type, by far the greater proportion of cases occur amongst the heads of families between the ages of 20 and 30, the very period when they have generally the greatest number of young children dependent on them for support. The effect of the physical causes of disease is not confined to any class or age."

After alluding to the decrease of mortality in improved districts; the charges upon the community from excessive disease; the influence of excessive mortality on the increase of population; the general deficiency in the supplies of water, and the practice of administering opiates to children, which, it is said, has "become to an alarming degree prevalent, especially in the manufacturing counties," the commissioners propose the following "remedial measures," in each of which they subsequently proceed more in detail to state their reasons, and such observations as had occurred to them on each branch of the subject.

"We are of opinion that, for the effectual correction of the evils above adverted to, additional legislative measures are requisite.

"It is necessary that the Crown should have power to inspect and supervise the execution of all general measures for the sanatory regulations of large towns and populous districts.

"That the local authorities entrusted with the execution of such measures should be armed with additional powers, and that the districts placed under their jurisdiction should in many cases be enlarged, and made co-extensive with the natural areas for drainage.

"We recommend, that the necessary arrangements for drainage, paving, cleansing, and an ample supply of water, (the most important matters conducive to health) should be placed under public inspection and control.

"The mode in which we propose to carry out these objects, is detailed in the Recommendations which are subsequently stated in this Report, with the reasons which have induced us to adopt them.

"We have arranged the different branches of the subject in the following order:

"1. Drainage, including house and main drainage, and the drainage of any space not covered with houses, yet influencing the health of the inhabitants.

"2. The paving of public streets and courts and alleys.

"3. Cleansing; comprising the removal of all refuse matter not carried off by drainage, and the removal of nuisances.

"4. A supply of water for public purposes and private use.

"5. The construction and ventilation of buildings for promoting and securing the health of the inhabitants."—*Second Report*, p. 7.

On all these topics, the testimony of competent observers, in and out of the medical profession, is given in detail; and every enlightened individual must be struck with the general pertinency of the questions and answers. For these the Report itself must be consulted, which will scarcely be found in many private libraries; but ought assuredly to be possessed by every one to which the public can have access, and by all municipal bodies. Amongst the medical gentlemen examined, were Drs. C. J. B. Aldis, Neill Arnott, W. A. Guy, E. Rigby, T. Southwood Smith, and Robt. Willis; and Mr. Toynbee,—all names familiar to our readers. The Commissioners were the Duke of Buccleuch and Queensberry, the Earl of Lincoln, Messrs. Harvey and Graham, Sir Henry Thomas de la Beche, Drs. Lyon Playfair and David Boswell Reid; Richard Owen, Esquire, Hunterian Professor of the College of Surgeons; Captain William Thomas Denison; Messrs. James Renald Martin, James Smith, Robert Stephenson, Jr., and William Corbett; and the commission empowers them to inquire "into the present state of large towns and populous districts in England and Wales, with reference to the causes of disease among the Inhabitants, and into the best means of promoting and securing the Public Health under the operation of the laws and regulations now in force, and the usages at present prevailing with regard to the drainage of lands, the erection, drainage and ventilation of buildings, and the supply of water in such towns and districts, whether for purposes of health, or for the better protection of property from fire, and how far the public health and the condition of the poorer classes of the people of this Realm, and the salubrity and safety of their dwellings, may be promoted by the amendment of such laws, regulations and usages."

But we cannot particularize further.

It yet remains to say a few—and we have only space for a few—words on the other works.

The Hygiène of Levy is one of the best that have appeared from the French press. There is none, indeed, so completely up to the day in the mode in which the matters belonging to hygiène are treated. The failing of his countrymen is, however, again observable. His knowledge is wholly French,—at least, he evidently has received what he knows of the doings abroad amongst "outside barbarians," almost wholly—if not wholly—through the medium of that language. Still, his book is richly worthy of being placed in the library of the scientific physician.

The very title of Dr. Metcalfe's book is enough to excite alarm in these sweltering dog-days. We have already expressed a general opinion of its merits. It is one of its leading objects, as Dr. Metcalfe states in his preface, to prove, by a careful generalization of facts, that caloric and electricity are mutually convertible; and "consequently that they are modifications of one and the same essence, which is the active principle in light, and in all the phenomena of nature." p. xii. The author's main views were first promulged in an Essay, entitled "A New Theory of Terrestrial Magnetism," published at New York in 1833, and somewhat extended in a series of papers in the Knickerbocker Magazine of 1834—5. The second volume contains the application of those views to Biotics—the science of life—and it furnishes many interesting materials for thought, into which we cannot at present enter. Should the work be reprinted here, opportunity may occur for a further notice.

The Pennsylvania Journal of Prison Discipline and Philanthropy is "devoted to the exposition and promulgation of correct views on Prison Discipline, Police Systems, Asylums for the Insane Poor, Societies for the aid of discharged prisoners, and other reforms immediately connected with those abuses." It is of course the great exponent of the "Pennsylvania" or "Separate System;" and so long as it is only the liberal exponent of that system, and does not enter into recriminatory polemical discussions, it does well. The "system" has been both misunderstood and misrepresented; but late occurrences seem to show, that it is about to be properly appreciated, and to be univeraally regarded as one of the great moral improvements of the age.

Hitherto the articles in the Journal have been generally written in good taste; and the work has been creditable to the active philanthropists who are concerned in its management. Harsh references to individuals—no matter what may be the provocation—should, if possible, be avoided; and under this view, we should have been glad if the allusion *ad hominem* in the following, not—by the way—most perspicuous passage, had been omitted:

"In all the prisons on the Auburn plan, numerous pardons are granted to those who are ill, and who would die if kept in confinement, in order to save life. This is avowed in a number of reports; and where it is not confessed in relation to others, we can prove it, if required, by any responsible person—the denial of Mr. Dwight, if it should be made, will not be regarded." p. 277.

We regard it as an error in this third number, that so much space is occupied by the memorial soliciting a State Hospital for the Insane, submitted by Miss Dix to the Legislature of Pennsylvania, Feb. 3, 1845, which, with the act to establish an asylum for the Insane Poor, occupies no less than 49 pages. Then follow an article by Dr. Emerson, on the mortality among the colored population of Philadelphia; and a republication on the effects of secluded and gloomy Imprisonment on Individuals of the African variety of mankind in the production of Disease, by Dr. B. H. Coates, which was read before the American Philosophical Society at its centennial celebration in 1843, and was afterwards published in the Transactions of the Historical and Literary Committee of that Society. Interesting as these papers are, they require no further notice from us at this day.

Some valuable Tables of Diseases and Deaths in the County Prison of Philadelphia follow; and there is also a communication on the ventilation and warming of Prisons and other Buildings, which may be perused with interest. It will be concluded in the next number.

On the whole, there is much matter of hygienic importance in the Journal; and should it continue to be conducted with spirit and ability, it cannot fail to be a valuable contribution to "philanthropy," and therefore ought eminently to receive favour from the medical profession, in whom science and philanthropy should be intimately blended; otherwise its members are but little adapted for their elevated avocation.

